# The influence of ego-involving climates on perceived competence and commitment for U.S. Masters swimmers

**DOI:** 10.3389/fpsyg.2025.1574429

**Published:** 2025-05-09

**Authors:** Troy O. Wineinger, Mary D. Fry, Haiying Long, Theresa C. Brown

**Affiliations:** ^1^School of Teaching, Learning, and Curriculum Studies, Kent State University, Kent, OH, United States; ^2^Department of Educational Psychology, University of Kansas, Lawrence, KS, United States; ^3^School of Nursing and Health Studies, University of Missouri Kansas City, Kansas City, MO, United States

**Keywords:** adults, seniors, achievement goal perspective theory, physical activity, motivational climate

## Abstract

**Introduction:**

The climate adults experience in their sport and physical activity endeavors may be central for them staying active and promoting healthy aging. Researchers have focused on the adaptive effects of the task-involving climate on adults’ sport experiences, though little attention has been given to the maladaptive influences of an ego-involving climate.

**Methods:**

The purpose of this study was to assess the relationship between Masters swimmers’ perceptions of an ego-involving climate, competence, and commitment, as well as investigate the moderating effect of perceived competence on Masters swimmers’ commitment within ego-involving climates. U.S. Masters swimmers (*n* = 566; Mage = 54.82; White 73.70%; female 67%) competing in coach-led programs completed an online survey.

**Results:**

Latent moderated SEM analyses revealed that Masters swimmers’ perceptions of an ego-involving climate did not predict their sport commitment, although the interactive effect of an ego-involving climate and perceived competence was significant for commitment. Conditional effects further revealed that while athletes with higher perceptions of competence showed a positive relationship between ego-involving climate and sport commitment, a stronger negative association was observed for Masters swimmers with lower perceptions of competence in an ego-involving climate.

**Discussion:**

Adults, regardless of experience or expertise, can benefit from participating in physical activity and avoiding ego-involving tendencies is essential to fostering their commitment to staying active.

## 1 Introduction

The worldwide population is aging, with the percentage of older adults (aged 60 years or over) increasing from 9.2% in 1990 to an estimated 18% in 2024, and projected to continue growing, reaching 24% of the global population by 2050 (United Nations, Department of Economic and Social Affairs, Population Division, 2023). Physical activity has proven beneficial for healthy aging throughout the lifespan and vital for adults to continue engaging in fundamental skills required to live independently (Edemekong et al., 2023). Beyond supporting activities of daily living, physical activity fosters the basic psychological needs (i.e., competence, relatedness, and autonomy); enhances mobility; reduces falls, disease, and illness risk; and overall promotes quality of life and longevity (Stehr et al., 2021; Stavrinou et al., 2022; Wickramarachchi et al., 2023). Despite the known benefits, approximately 80% of U.S. adults do not meet recommended physical activity guidelines for optimal health (Elgaddal et al., 2022). Supporting adults’ physical activity participation is essential, as even individuals who meet the guidelines may discontinue if they perceive a lack of support (Burton et al., 2017). Time constraints and lack of motivation are common factors contributing to attrition, prompting researchers to call for a deeper understanding of behavioral constructs that may prevent physical activity withdrawal (Collins et al., 2022; Martínez-Ramos et al., 2015; Stehr et al., 2021).

Master athletes are an ideal group to gain valuable insights into promoting physical activity among adults, recognized for their exemplary exercise participation. Master athletes are individuals aged 35 and older who demonstrate high levels of consistent participation in physical activity through competitive sport, often exhibiting the physical, mental and social benefits of sustained physical activity to longevity and quality of life (Bortz and Bortz, 1996; Geard et al., 2017). While most physical activities are beneficial, swimming has grown increasingly popular among adults for improving aerobic fitness and building muscle due to its lower impact on knees and hips (Cooper et al., 2007). The decision whether to engage and persist in sport and physical activity is multi-factorial (Selejó Joó et al., 2024), and athletes will have varying levels of psychological factors (e.g., motivation, enjoyment) and external factors (e.g., social support, access to facilities), which can all play critical roles in their commitment (Bauman et al., 2012). Specific to motivation, Masters swimmers can be motivated by both intrinsic factors (e.g., enjoyment) and extrinsic factors (e.g., competition) contributing to their long-term physical activity participation (Brilliant et al., 2021), with adults more likely to prioritize health and well-being over other fitness goals as key drivers to their commitment (Molanorouzi et al., 2015).

Understanding the antecedents that promote sustained engagement in physical activity is essential for promoting adults’ long-term participation. Achievement goal perspective theory (AGPT; Nicholls, 1984, 1989) provides a valuable framework to foster adaptive outcomes promoting physical activity engagement. A central tenet of AGPT is the motivational climate, where leaders are encouraged to cultivate a task-involving climate that values effort, improvement, cooperation, and learning from mistakes while minimizing ego-involving climate tendencies that focus on normative comparison, rivalry, and recognition of only the highest performers. Research consistently supports the benefits of a task-involving climate (Fry and Moore, 2019). For example, Fry et al. (2023) found that Masters swimmers’ perceptions of a task-involving climate were linked to greater effort, enjoyment, and flourishing.

Despite these benefits, ego-involving climates persist across physical activity contexts for all age groups, leading motivational researchers to raise concerns about their negative impact, particularly for those with low perceived competence (Duda and Whitehead, 1998; Roberts, 2012). Nicholls (1984, 1989) emphasized the role of the motivational climate in shaping individuals’ perceptions of competence and predicting individuals’ affective, cognitive, motivational, and behavioral responses. While the task-involving climate is universally adaptive, regardless of individuals’ perceptions of competence, low perceived competence is particularly problematic in an ego-involving climate where emphasis is on comparison to others and success is based on a normative standard (Ames, 1992; Nicholls, 1992). In an ego-involving climate, individuals with low perceived competence are more likely to struggle, feel inadequate, and experience frustration or discouragement (Ingrell et al., 2016; Roberts, 2012; Sarrazin et al., 2002; Wu et al., 2024; Yoo, 2003). As individuals are vulnerable to feelings of failure in comparison-based settings, they may experience declining motivation as enjoyment decreases, undermining commitment to long-term participation in the activity (Granero-Gallegos et al., 2017; Jõesaar et al., 2011; MacDonald et al., 2011; Smith et al., 2008).

While much of the research including the ego-involving climate has been conducted in youth sport settings, early evidence suggests the potential negative impact of an ego-involving climate persists in adult physical activity settings as well. For example, researchers have found the ego-involving climate was associated with lower commitment to exercise among college students 18 years and older (Brown and Fry, 2013). Ego-involving climates have been associated with negative physiological and psychological effects, such as increased inflammation and inhibiting basic psychological needs, illustrating the detrimental effects when comparison and competition overshadow personal progress and effort (Hogue, 2024). Jo (2022) demonstrated older adults (60 years and older) experienced greater competence, well-being, and commitment to exercise in group fitness classes where ego-involving tendencies were minimized. Similarly, Santi et al. (2014) found Masters swimmers in ego-involving climates showed lower sport commitment and participation, suggesting that such climates can reduce long-term engagement in the activity. Despite these findings, the adult population spans a wide age range, and further research is needed to fully recognize the impact in different physical activity settings.

This study addresses a gap in the current literature by examining the role of an ego-involving climate specifically within the context of Master athletes competing in their sport, a population that is often underrepresented in research on physical activity. Building on AGPT’s insights into physical activity and the established negative relationship between an ego-involving climate and motivational responses, the primary purpose of this study was to examine the relationship between Masters swimmers’ perceptions of an ego-involving climate, competence, and commitment. Masters swimmers’ perceptions of competence were expected to be positively associated with their commitment to continue swimming, with no significant association with their perceptions of an ego-involving climate. The secondary purpose of this study was to explore how perceived competence moderated the relationship between Masters swimmers’ perceptions of an ego-involving climate and their sport commitment. The hypothesis was that highly competent athletes would exhibit greater commitment to their sport in a high (vs. low) ego-involving climate. However, athletes with low competence would show lower commitment to their sport in a high (vs. low) ego-involving climate. Understanding how ego-involving climates influence older adults’ commitment to sport is crucial for developing strategies that encourage sustained participation in physical activity and promote long-term health benefits.

## 2 Materials and methods

### 2.1 Participants

U.S. Masters swimmers (*N* = 566; female 67%) were invited to complete a brief survey to provide feedback on their swim experiences in the U.S. Masters Swimming (USMS) organization. USMS is a national organization providing adults with the opportunity to participate in organized swim programs, competitions and events and promotes fitness and camaraderie through swimming for adults of all skills levels. Only Masters swimmers who were 35 years and older (*N* = 479; *M*age = 55.72; age range = 35–87; female 69.20%; White 94.70%; Hispanic 2.10%; Asian 1.90%) were included in the sample. This sample size was considered excellent to perform structural equation modeling (SEM), as the sample size recommended in a typical SEM analysis is 200 (Feng and Hancock, 2023; Kline, 2023).

### 2.2 Procedure

The Institutional Review Board at the lead author’s university approved this study. The anonymous Qualtrics survey was distributed by USMS administrators to all members who fit the identified qualifications. Those agreeing to participate were required to complete an informed consent. The survey included self-reported measures of Masters swimmers’ perceptions of the ego-involving climate, commitment to their sport, perceived competence in swimming, and demographic information (i.e., age, race).

### 2.3 Measures

#### 2.3.1 Ego-involving climate

Masters swimmers’ perceptions of the ego-involving climate in their swim environments was assessed using the 6-item ego-involving climate scale of the Perceived Motivational Climate in Exercise Questionnaire—Abbreviated (Moore et al., 2015). The stem for the items was “At my swim facility…” and a sample item is “Coach(es) give most of their attention to only a few Masters swimmers.” Masters swimmers responded to items on a five-point Likert scale ranging from 1 (Strongly Disagree) to 5 (Strongly Agree). Moore et al. (2015) have supported the reliability (*α* = 0.72–0.80) of this scale with three different adult samples in the exercise setting (i.e., international exercise franchise, university physical activity classes, campus recreation center).

#### 2.3.2 Sport commitment

The 4-item sport commitment subscale of the Sport Commitment Model (Scanlan et al., 1993) was utilized to evaluate Masters swimmers’ commitment to swimming. Although established in the youth baseball and softball setting, the measure is readily adapted to the swimming context. A sample item is “How determined are you to keep swimming?” Masters swimmers responded using a five-point Likert scale ranging from 1 (Not At All/Nothing) to 5 (Very Dedicated/Very Hard/Anything). Alexandris et al. (2002) found support for the reliability (*α* = 0.86) and validity of this instrument with adult exercise and fitness participants using Confirmatory Factor Analysis (CFA).

#### 2.3.3 Perceived competence

The 5-item competence subscale of the Intrinsic Motivation Inventory (McAuley et al., 1989) was employed to measure Masters swimmers’ perceptions about their abilities with respect to swimming. Although established with undergraduate students enrolled in physical activity courses, the measure is readily adapted to the swimming context. A sample item is “I feel pretty competent at swimming.” Masters swimmers responded using a five-point Likert scale ranging from 1 (Strongly Disagree) to 5 (Strongly Agree). McAuley et al. (1989) have provided CFA and reliability (α = 0.80) support for using this measure in college physical education classes. Additionally, Huddleston et al. (2012) have provided support for the reliability of the measure (α = 0.84) with adult fitness members.

### 2.4 Data analyses

Before the main analysis, descriptive statistics and reliabilities of all measures were calculated and the data was assessed for data normality (i.e., skewness and kurtosis) and missingness in IBM SPSS version 29 (IBM Corp., 2021). The skewness of all variables ranged between −1.62 and 1.13 and kurtosis ranged between −1.86 and 2.46. These skewness and kurtosis values were within the normality range for a sample that is larger than 300 (Mishra et al., 2019). There were 5.43% of missing values in all observations. Little’s (2013) MCAR (Missing Completely At Random) test yielded a nonsignificant result (χ2 _(320)_ = 350.37, *p* = 0.12), suggesting that the missing values were missing completely at random. Latent moderated Structural Equation Modeling (SEM) analysis was used, which is a sophisticated statistical method incorporating the examination of measurement models and structure models simultaneously to analyze the moderation relationships among latent variables (Kline, 2023). Latent moderated SEM analysis allows researchers to test whether the relationship between an exogenous variable (i.e., ego-involving climate) and an endogenous variable (i.e., sport commitment) is influenced by a moderator (i.e., perceived competence), after controlling the measurement errors. The analysis was conducted in M*plus* 8.11 using an iterative maximum likelihood estimation procedure in the latent moderated SEM (Muthén and Muthén, 1998–2012).

The measurement model was tested first using CFA to support the construct validity of all the latent variables (Anderson and Gerbing, 1988). Then, the interaction between ego-involving climate and perceived competence was assessed using three methods. First, the fit indices of log-likelihood, Akaike Information Criteria (AIC), Bayesian Information Criteria (BIC), and sample-size adjusted BIC were produced both without the interaction (Model 0) and with the interaction (Model 1). Smaller values in AIC and BIC indicate better fit, while a statistically significant log-likelihood ratio test suggests a better fit between the two models. It is important to note the latent moderated SEM does not produce the same fit indices used in other SEM analyses (e.g., mediation) such as Comparative Fit Index (CFI), Tucker-Lewis Index (TLI), Root Mean Square Error of Approximation (RMSEA), and Standardized Root Mean Square Residual (SRMR); instead, it produces log-likelihood, AIC, BIC, and sample-size adjusted BIC. Second, the variance explained by the interaction was examined as the effect size. Third, the interaction effect is visualized in a plot to help interpret the relationship between the exogenous and endogenous variables by using pick-a-point strategy (Rogosa, 1980), in which the moderator was estimated at a higher level (mean plus 1 standard deviation) and a lower level (mean minus 1 standard deviation) (Béland et al., 2022). Additionally, all the indicators of the exogenous variable were standardized and used ALGORITHM = INTEGRATION syntax to estimate latent variable distributions. Full information maximum likelihood (FIML) was implemented to account for missing data in the analysis (Enders and Bandalos, 2001), and 95% confidence intervals were calculated.

## 3 Results

### 3.1 Descriptive statistics and correlations

The majority of Masters swimmers reported perceiving a moderately ego-involving climate within their swimming environment and overall high sport commitment and perceived competence (Table 1). The Pearson correlations indicated that having higher sport commitment was associated with Masters swimmers reporting greater perceived competence.

**Table 1:** Means, standard deviations (SD), minimum, maximum, Cronbach alpha (*α*), and Pearson correlations.

Measure	*M*	SD	Min	Max	α	1	2	3
1. Ego-involving climate	2.31	0.63	1.00	4.50	0.75	1		
2. Perceived competence	3.98	0.64	1.00	5.00	0.87	−0.06	1	
3. Commitment	4.36	0.50	1.75	5.00	0.80	−0.06	0.27**	1

All measures utilized a 1 to 5 Likert scale. *p < 0.05; **p < 0.001.

### 3.2 CFA

**Figure 1:**
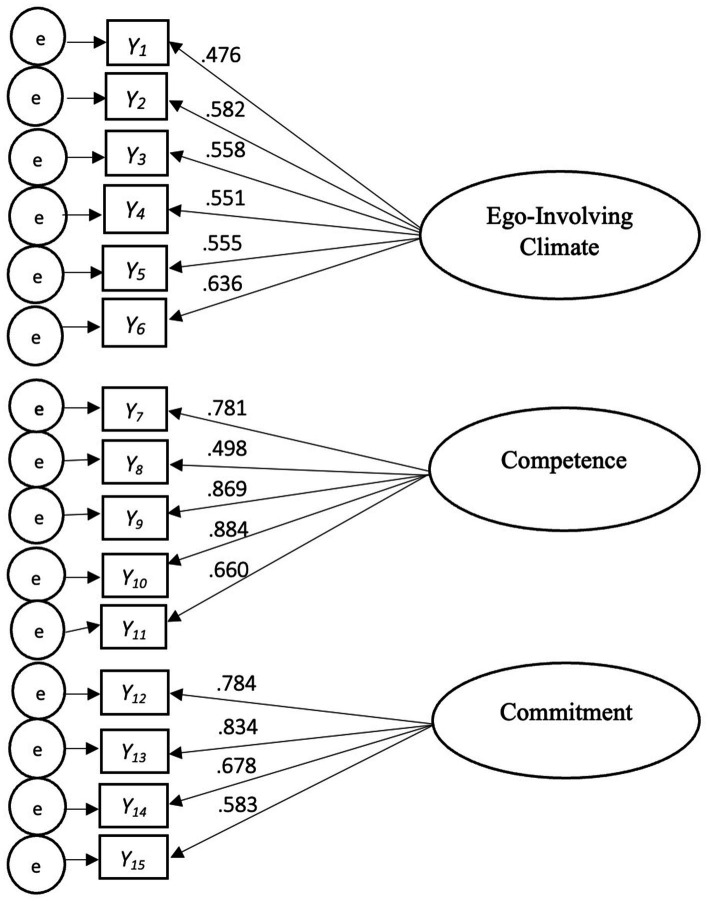
Final measurement model.

The original CFA of all the measures showed adequate model fit [CFI = 0.92, TLI = 0.90, RMSEA = 0.06 (90% CI: 0.055, 0.07), SRMR = 0.05]. Based on the modification index suggested by M*plus*, the error variance of the sixth and seventh items of the ego-involving climate construct were correlated, yielding excellent model fit [CFI = 0.95, TLI = 0.94, RMSEA = 0.05 (90% CI: 0.042, 0.060), SRMR = 0.05]. Additionally, most factor loadings of the items for the latent variables fell between 0.53 and 0.89 (Figure 1).

### 3.3 Moderated SEM

**Figure 2:**
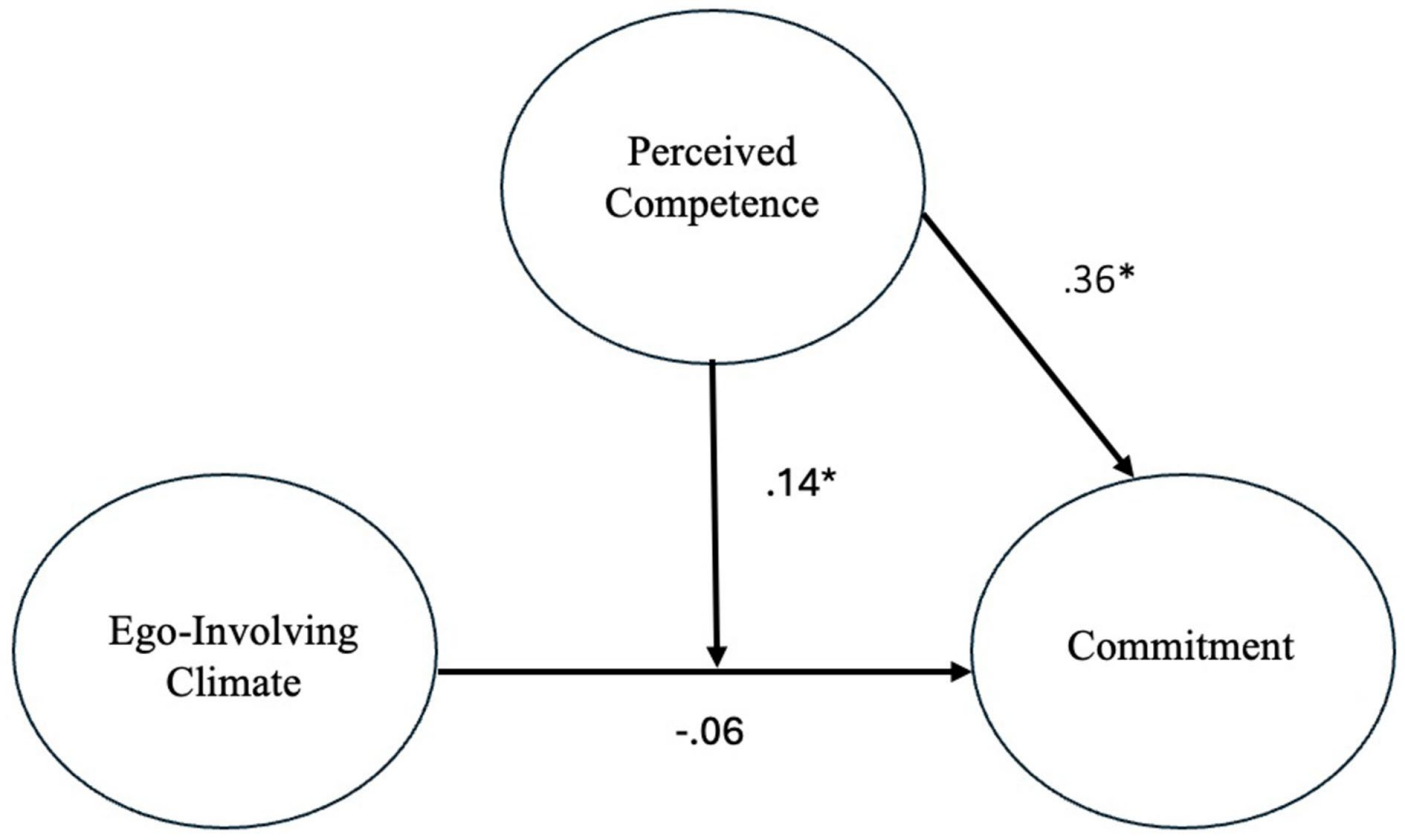
Moderation model. *p ≤ 0.05.

First, a latent moderated SEM analysis was performed with ego-involving climate (exogenous variable), perceived competence (moderator), and sport commitment (endogenous variable) (Figure 2). Results from the analysis indicated that perceived competence had a significant and strong relationship with sport commitment [*β* = 0.309, *p* < 0.001, 95% CI (0.217, 0.401)], while perceptions of an ego-involving climate had no significant relationship with sport commitment [*β* = −0.092, *p* = 0.186, 95% CI (−0.207, 0.022)]. Furthermore, the model with interaction had a slightly better AIC, BIC, and sample-size adjusted BIC than the model without interaction (Table 2). The log-likelihood ratio test was not significant. Although the *p* value of the interaction between the two variables was 0.055, the 95% CI for the interaction effect was 0.019, 0.253, which did not include 0, suggesting a significant interaction effect between perceptions of an ego-involving climate and competence on sport commitment. The standardized coefficient of the interaction was 0.136, though the variance explained by the interaction was small (0.007).

**Table 2:** Model fit comparison.

Model	AIC	BIC	Sample-size adjusted BIC	Log-likelihood	Number of parameters	*R* ^2^
Model 0	15939.93	16152.69	15990.82	−7918.97	51	0.123
Model 1	15934.88	16151.80	15986.76	−7915.44	52	0.130
Difference	5.05	0.89	4.06	−3.53	1	0.007

Model 0 is without interaction and model 1 is with interaction.

**Figure 3:**
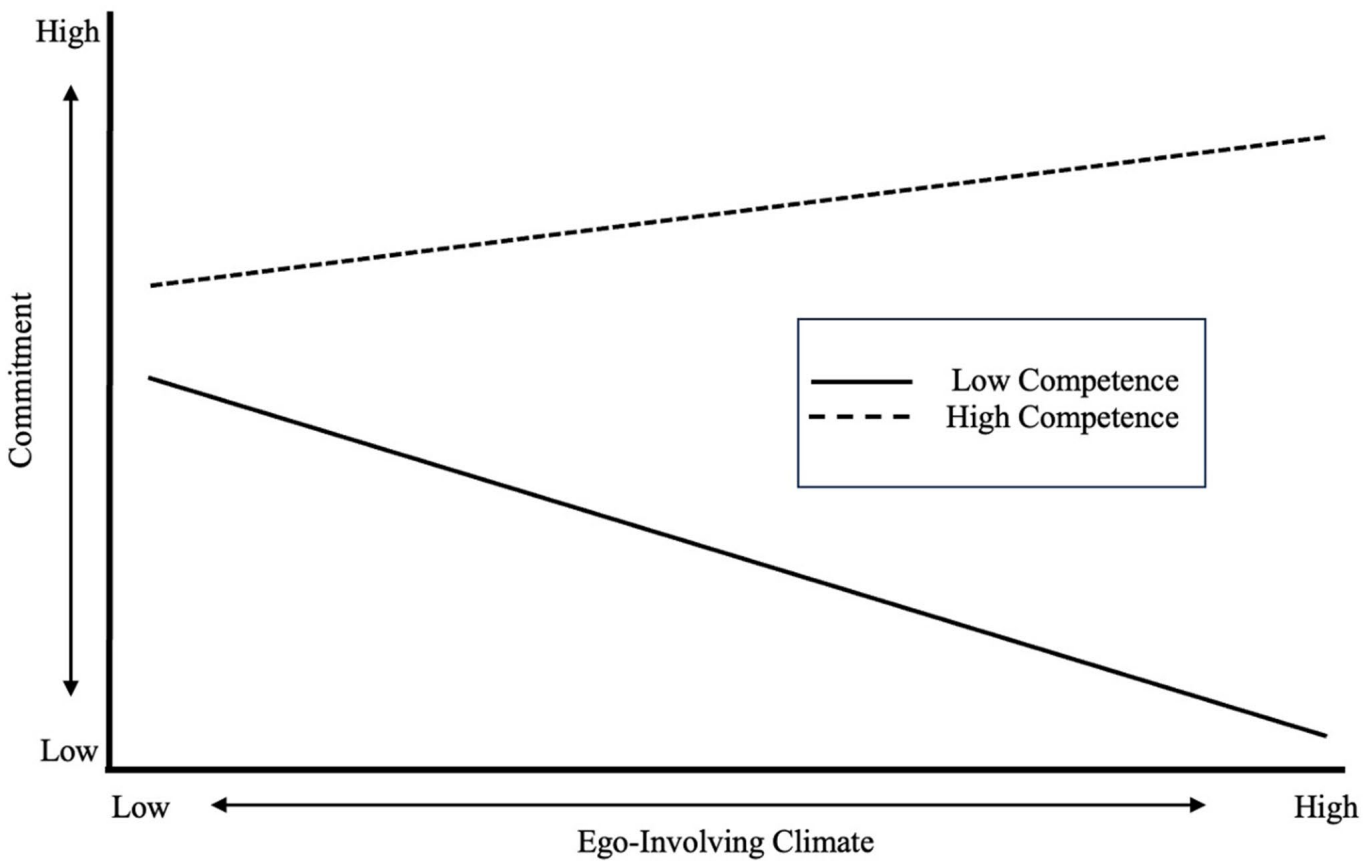
Relationship between commitment and perceived competence when in an ego-involving climate.

Further conditional effects were analyzed for the moderation analysis based on two groups with high and low levels of perceived competence, which were determined by being one standard deviation above and below the mean level of perceived competence, respectively. There was a positive relationship between being in an ego-involving climate and sport commitment for Masters swimmers with higher perceptions of competence, though the relationship was not significant (*b* = 0.102, *p* = 0.213). In contrast, a significant negative relationship was found between Masters swimmers’ perceptions of an ego-involving climate and sport commitment for Masters swimmers with lower perceptions of competence (*b* = −0.245, *p* = 0.048). The plot showed the same trend (Figure 3), with a positive relationship between the ego-involving climate and sport commitment for athletes with higher perceptions of competence; and a negative relationship for athletes’ sport commitment when they had lower perceptions of competence.

## 4 Discussion

The primary purpose of this study was to assess the relationship between Masters swimmers’ perceptions of an ego-involving climate, sport commitment, and their perceived competence. As hypothesized, Masters swimmers who reported higher levels of sport commitment also reported greater sport competence, whereas perceptions of the ego-involving climate were not significantly associated with their competence or sport commitment. Additionally, the moderating role of competence between the ego-involving climate and sport commitment was examined. For those perceiving an ego-involving climate, lower competence was associated with less sport commitment and those with greater competence reported greater sport commitment to swimming. The significant interaction effect, indicated by the confidence interval not including zero, demonstrated perceived competence influenced how perceptions of the ego-involving climate impacted sport commitment. These findings are consistent with theoretical predictions and existing AGPT research, emphasizing the nuanced role perceived competence plays in the relationship between the ego-involving climate and long-term commitment to physical activity (Brown and Fry, 2014; Granero-Gallegos et al., 2017; Ingrell et al., 2016; Nicholls, 1984, 1989; Wu et al., 2024).

Nicholls (1984, 1989) theorized that in an ego-involving climate, competence would be undermined for a large portion of the group, as only those with the best normative performances are valued and recognized. The current study findings suggest an ego-involving climate may be particularly detrimental with Masters swimmers who have low competence in their swim abilities. Noteworthy was the steeper slope for the low perceived competence group indicating a sharper decrease of sport commitment in the ego-involving climate. Santi et al. (2014) have found that supportive teammates and coaches play a key role in fostering sport commitment among Masters swimmers. The current results further support these recommendations, suggesting professionals supporting adult sport and physical activity endeavors should avoid creating an ego-involving climate, especially when perceived competence levels may not be known and the goal is to promote long-term engagement.

While one may conclude from the current results an ego-involving climate might be advantageous for athletes with high perceived competence, such a conclusion is problematic for several reasons. First, survey measures capture perceived competence at one time point, yet perceptions of competence can fluctuate as individuals navigate different challenges and experiences over time (Liem, 2022). These fluctuations to perceived competence are particularly salient with adults, as aging can affect competence through factors like declining health, reduced performance, increased risk of falls or injuries, and the likelihood of competing with others who may perform better (Overdorf et al., 2016).

Second, the goal in applied motivational climate research is to optimize motivation and participation in physical activity for all individuals, regardless of their perceived competence. When a climate emphasizes normative comparisons, rivalry, and recognition based solely on top performers, it risks alienating those with lower perceived competence. In contrast, a task-involving climate, which emphasizes effort, progress, and personal growth, benefits both high and low-competence participants, fostering a more inclusive and sustainable environment where everyone is encouraged to continue participating. Social comparisons can be a deterrent for adults in exercise contexts, especially if they fear appearing incompetent among their peers (Kappen et al., 2016). Ego-involving climates naturally promote these types of comparison, something avoidable in a task-involving approach (for review, see Fry and Moore, 2019, Harwood et al., 2015).

Third, maximizing adults’ sport commitment to live a physically active lifestyle is critical to promote healthy aging across the lifespan (Edemekong et al., 2023). To encourage consistency, all active adults should be recognized for their effort and commitment, with greater focus on what they can control, rather than spotlighting the best performers at any given time. As the populations of the United States and world continue to age (Caplan, 2023; United Nations, Department of Economic and Social Affairs, Population Division, 2023), healthy aging is especially critical. Physical inactivity costs the worldwide health-care system billions annually, with a particularly severe impact in wealthier countries such as the U.S. (Ding et al., 2016; Tang et al., 2022). Physical inactivity is largely preventable, and any intentional approach to encourage more movement is needed to promote movement as a means to improve quality and quantity of life (Santos et al., 2023).

Drawing from previous studies, specific coaching interventions aimed at reducing the ego-involving climate for Master athletes can be effectively delivered through targeted coach education programs that focus on fostering a task-involving climate. For example, while specific to youth, the Promoting Adolescent Physical Activity (PAPA) project (Duda, 2013) and coaching interventions in community-based sport leagues (Smith et al., 2007) demonstrate that short, focused educational interventions can significantly alter coaches’ motivational approaches. Likewise, Claunch and Fry (2016) designed a coaching intervention to help Native American college football coaches implement more task-involving characteristics into their coaching style, leading coaches to modify their behaviors during practices and games. While these examples are specific to a younger age cohort, coaching programs for Master athletes could provide education on how to create a climate where athletes are motivated by personal growth and enjoyment rather than external validation or competition. To support the coach training, the coaching programs could adopt structured psychological safety measures, such as regular check-ins and feedback opportunities, setting clear expectations, celebrating effort over outcome, and providing supportive and constructive feedback. By incorporating these principles, coaching staffs could help Masters athletes improve their performance, and experience continued enjoyment and longevity in their sport.

## 5 Limitations and future direction

The findings highlight the negative impact of an ego-involving climate on adult sport participants, supported by a large sample size and robust moderation model. Despite these strengths, there are opportunities to advance the research further. The results of this study were based on a single timepoint, precluding any causality inference. The physiological consequences of being in an ego-involving climate suggest that the long-term effects of remaining in such an environment could be detrimental, as researchers have noted maladaptive physiological and psychological responses (Hogue, 2024). However, the specific changes and long-term effects associated with prolonged exposure to an ego-involving climate are not well known. Future research should explore experimental designs (e.g., qualitative) and multiple data collection points to better infer casual relationships between variables and examine long-term impact of athletes experiencing an ego-involving climate.

While the theoretical framework for this study focused on the impact of the ego-involving climate, future research might also consider how other identifiers moderate the relationship between motivational climate and commitment to sport. Previous work with Master athletes has suggested age is not related to athletic commitment and rather, other drivers impact commitment and performance such as intrinsic motivation, social support, improved health benefits, and habit (Stones, 2019). These factors could act as additional moderators explaining the relationship between the motivational climate and sport commitment as well as other considerations such as prior competitive experiences or whether Masters swimmers are currently competing. To that end, future researchers would benefit by investigating experiences of both athletes and coaches through qualitative studies.

Lastly, the present study focused solely on the impact of an ego-involving climate on Masters swimmers’ commitment to the sport, and there is opportunity for additional outcome variables such as goal orientations, motivational responses (e.g., worry, anxiety, burnout), and enjoyment among adult populations. High ego orientation is often linked to maladaptive responses such as worry, anxiety, burnout, decreased effort, and less enjoyment (Fry and Moore, 2019). Newton and Fry (1998) found that senior Olympians’ high in task orientation had higher intrinsic motivation and believed success was driven by hard work. A high task orientation may buffer the effects of low competence, helping individuals maintain intrinsic motivation and commitment, whereas those with a high ego orientation might experience stress in an ego-involving climate, where performance outcomes dominate.

## 6 Conclusion

Overall, this study contributes to the AGPT and sport psychology literature by highlighting the impact of ego-involving climates, particularly among adults with low perceived competence. Given the importance of physical activity for optimizing physical and mental health across the lifespan, coaches should be cautious when fostering highly ego-involving team environments. This research illustrates how such climates may undermine the experiences of Masters swimmers, particularly those who feel less competent. The findings are both novel and impactful, replicating similar results observed in younger sport and exercise participants (Sarrazin et al., 2002; Smith et al., 2008), while expanding our understanding of adults’ sport experiences, which have been less studied. Of particular note is the demonstrated moderating effect of perceived competence on adults’ sport commitment within an ego-involving climate. This research can be used to enhance adults’ physical activity experiences, which is crucial for promoting sustained movement throughout the lifespan.

## Data Availability

The raw data supporting the conclusions of this article may be made available by the authors, without undue reservation.
